# Specific oligonucleotide primers for detection of endoglucanase positive *Bacillus subtilis* by PCR

**DOI:** 10.1007/s13205-013-0177-6

**Published:** 2013-09-28

**Authors:** S. Ashe, U. J. Maji, R. Sen, S. Mohanty, N. K. Maiti

**Affiliations:** Division of Fish Health Management, Central Institute of Freshwater Aquaculture, Kaushalyaganga, Bhubaneswar, 751002 Orissa India

**Keywords:** Endoglucanase gene, PCR, Specific primers, *B. subtilis*

## Abstract

A polymerase chain reaction (PCR) assay was developed for discrimination of *Bacillus subtilis* from other members of *B. subtilis* group as well as rapid identification from environmental samples. Primers ENIF and EN1R from endoglucanase gene were used to amplify a1311 bp DNA fragment. The specificity of the primers was tested with seven reference strains and 28 locally isolated strains of endoglucanase positive *Bacillus* species. The PCR product was only produced from *B. subtilis*. The results demonstrated high specificity of two oligonucleotides for *B. subtilis*. This species-specific PCR method provides a quick, simple, powerful and reliable alternative to conventional methods in the detection and identification of *B. subtilis*. To our knowledge this is the first report of a *B. subtilis* specific primer set.

## Introduction

Genus *Bacillus* is a Gram-positive, spore-forming, fermentative, aerobic and rodshaped bacteria. Several species of this group are non-pathogenic, simple to cultivate and secrete enzymes such as proteases, amylases and cellulases that are useful for a number of industrial applications (Arbige et al. 1993). The *Bacillus subtilis* group contains the closely related taxa *Bacillus subtilis* subsp. *subtilis* (Smith et al. 1964; Nakamura et al. 1999), *Bacillus licheniformis* (Skerman et al. 1980), *Bacillus amyloliquefaciens* (Priest et al. 1987), *Bacillus atrophaeus (*Nakamura 1989), *Bacillus mojavensis* (Roberts et al. 1994), *Bacillus vallismortis* (Roberts et al. 1996*), Bacillus subtilis* subsp. *spizizenii* (Nakamura et al. 1999). Classical identification methods based on biochemical tests or fatty acid methyl ester profiling were laborious and hence not applicable for the purpose of a rapid screening. These taxa can be differentiated from one another by fatty acid composition analysis, restriction digest analysis and DNA–DNA hybridization analysis, but are quite difficult to differentiate by phenotypic characteristics (Roberts et al. 1994; Nakamura et al. 1999). The use of the 16S rRNA sequence as a target for genetic detection was therefore considered. Numerous *Bacillus* species described so far have been found to display rather conserved 16S rRNA sequences compared to other genera. Thus the use of this taxonomic marker is sometimes inadequate for species definition according to generally accepted criteria (Stackebrandt and Goebel 1994). Such unusual similarities exist for members of the ‘*Bacillus* 16S rRNA groupI’, including *B. subtilis*, which displays 99.3 % similarity at the 16S rRNA level to *B. atrophaeus* and 98.3 % to *B. licheniformis* and *B. amyloliquefaciens* (Ash et al. 1991). In the present study, it has been shown that specific primers for detection of endoglucanase gene could be used for identification of *B. subtilis.*

## Materials and methods

### Bacterial strains and culture conditions

A total 35 *Bacillus* strains were used in this study (Table 1). Of 12 *B. subtilis*, ten strains were isolates from pond sediments. For *Bacillus cereus,* one ATCC strain and nine pond sediment isolates were tested. Five pond isolates that had been classified as *Bacillus pumilus* and two as *B. amyloliquefaciens* were also included in the test. For *Bacillus megaterium,B. licheniformis* and *Bacillus thuringiensis,* ATCC strains were analysed. All the pond sediment isolates were identified by 16S rDNA sequencing and available in our laboratory.

**Table 1:** Bacterial strains used

Species	Total no. of strains	Source	Accession number of sediment isolates
*Bacillus subtilis*	14	ATCC 11,774, ATCC 6,051 and 12 pond sediment	GQ214130, GQ21413 HQ388810–HQ388813 JX438679–JX438684
*Bacillus cereus*	10	ATCC 13,061 and 9 pond sediment	GQ214131 HQ388814–HQ388817 JX438685–JX438788
*Bacillus pumilus*	6	ATCC 14,884 and 5 pond sediment	HQ388808 JX438694–JX438697
*Bacillus megaterium*	1	ATCC 9,885	
*Bacillus thuringiensis*	1	ATCC 10,792	
*Bacillus licheniformis*	1	ATCC 13,061	
*Bacillus amyloliquefaciens*	2	Pond sediment	JX438692–JX438693

Pond sediment isolates were confirmed by 16s rDNA sequencing

### Soil samples

24 Soil samples collected from agriculture field and fish culture ponds were also included.

### Primers

The endoglucanase gene sequences (EC, 3.2.1.4) of *B. subtilis* were retrieved from GenBank nucleotide database and were aligned using Clustal W (1.82) Multiple Alignment Program. Two sets of primers EN1F (103–124 bp) 5′-CCAGTAGCCAAGAATGGCCAGC-3′, EN1R (1,413–1,393 bp) 5′-GGAATAATCGCCGCTTTGTGC-3′) were designed by analyzing the conserved regions of the aligned sequences.

### DNA isolation

The total genomic DNA was extracted from bacterial suspension (after 12 h incubation in LB) using DNA extraction kit (Merck Bioscience, India) following the manufacture’s instruction. Soil genomic DNA was extracted by using ultra clean soil DNA isolation kits (MoBio, USA).

### Polymerase chain reaction

The PCR reaction mixtures (50 μl) contained, dNTPs each 100 μmol; 1X PCR buffer (10 mM Tris Cl, 50 mM KCl, 1.5 mM MgCl_2_ and 0.01 % gelatin); each primer 10 pmol; *Taq* DNA polymerase (NEB) 0.75U and bacterial DNA 100 ng. The touch down PCR in a volume of 50 μl was carried out with initial denaturation of 94 °C for 5 min followed by ten cycles of touch down program (94 °C for 30 s, 70 °C for 20 s and 74 °C for 45 s, followed by a 1 °C decrease of the annealing temperature every cycle). After completion of the touch down program, 25 cycles were subsequently performed (94 °C for 30 s, 60 °C for 20 s and 74 °C for 45 s) and ending with a 10 min extension at 74 °C. PCR reactions were run on a 1.5 % agarose gel in 1X TBE.

### Cloning and sequencing

Band was excised from the gel and PCR product was purified by using the QIAquick gel purification kit according to the manufacturer’s instructions (QIAGEN, Germany). The purified PCR product was cloned in pGEM^®^-T Easy vector following manufacturer’s protocol (promega) and transformed into DH5α cells. Sequencing of the positive clones were done by Sanger method using 96 capillary high through put sequencer; ABI 3,730 XL (Xcelris, India) with T7 and SP6 universal primer.

## Results and discussion

BlastN seach of endoglucanase gene of *B. subtilis* (accession numbers HM470252.1, AF355629.1 and CP002906.1) revealed 93 % similarity with *B. amyloliquefaciens, B. megaterium, B. pumilus* and *B. licheniformis* 90 % with *B. subtilis* subsp. *spizizenii* and 98–99 % with *Geobacillus stearothermophilus* and *Paenibacillus campinasensis.* Based on multiple alignments of endo-β-1,4-glucanase genes, a specific consensus motif was identified in the endoglucanase gene of *B. subtilis, G. stearothermophilus* and *P. campinasensis.* Two PCR primers, EN1F and EN1R, were chosen that were predicted to specifically amplify a 1,311 bpDNA fragment of the *B. Subtilis, G. stearothermophilus* and *P. campinasensis.* The Genbank database (NCBI) search for complimentary sequences revealed 100 % homology between the primers and the gene encodes endo-β-1,4-glucanase of *B. subtilis* as well a*s G. stearothermophilus* and *P. campinasensis.* No homologous sequences were found for other members of genus *Bacillus* indicating an excellent specificity of the primers for *B. subtilis.*

**Fig. 1:**
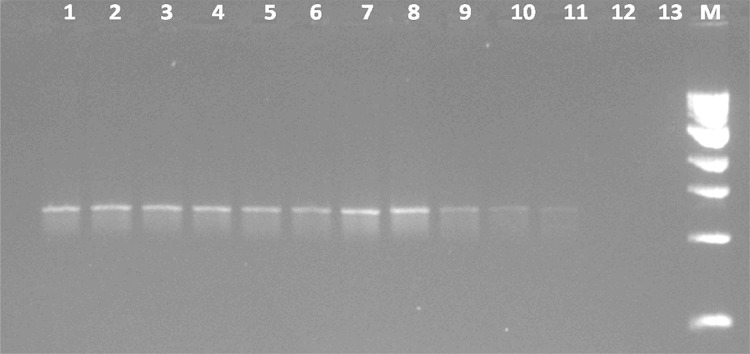
Limit of detection of endoglucanase gene in different concentration of DNA. *Lane 1* 100 ng, *lane 2* 50 ng, *lane 3* 10 ng, *lane 4* 5 ng, *lane 5* 1 ng, *lane 6* 100 pg, *lane 7* 50 pg, *lane 8* 10 pg, *lane 9* 5 pg, *lane 10* 1 pg, *lane 11* 500 fg, *lane 12* 100 fg, *lane 13* negative control, *lane M* size marker (1 kb ladder, NEB)

As expected, the test turned out to be positive only for *B. subtilis* among the different species of Genus *Bacillus* PCR amplification with genomic DNA isolated from in vitro cultured *B. subtilis* resulted in a reproducible amplification of 1,311 bp product with primer combinations EN1F/EN1R. To determine the sensitivity of PCR, endpoint titration with serial dilutions of genomic DNA isolated from the standard strain of *B. subtilis* was carried out and positive results obtained as little as 500 ficogram of DNA (Fig. 1).

To assess the range of specificity of the PCR test, a number of endoglucanase positive *Bacillus* species were assayed. Given the considerable number of species established to date under *B. subtilis* group, our choice to assess the range of specificity was restricted to *B. subtilis* subsp. *subtilis* that was representative of the *B. subtilis* group.

**Fig. 2:**
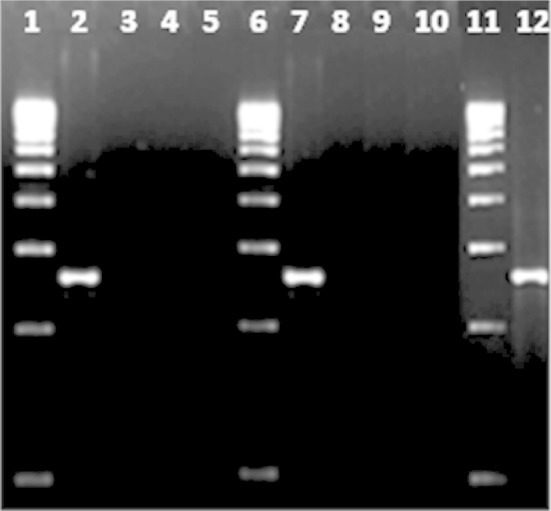
PCR amplification for endoglucanase gene in different *Bacillus* spp. *Lane 1* size marker (500 bp ladder); *lanes 2–5B. subtilis* ATCC-6,051, *B. cereus*-ATCC 13,061, *B. pumilus* ATCC-14,884, *B. megaterium* ATCC-9,885; *lane 6* size marker (500 bp ladder); *lanes 7–10B. subtilis* ATCC-11,774, *B. thuringiensis* ATCC-10,792, *B. licheniformis* ATCC-13,061, *B. amyloliquefaciens* CF8; *lane 11* size marker (500 bp ladder); *lane 12B. subtilis* C11B1

**Fig. 3:**
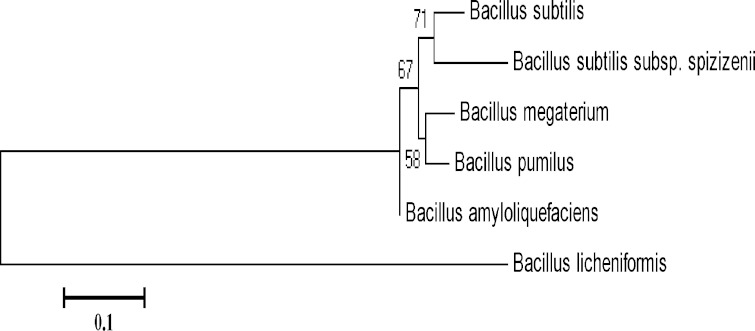
Phylogenetic tree of *Bacillus* spp. based on endoglucanase gene sequence data

To test the specificity of the amplified products, control experiments were performed under the same conditions with DNA from different members of *B. subtilis* group as well as *B. cereus* group. The test found to be positive only for *B. Subtilis* (Fig. 2). It is noteworthy that the species detected as positive with this test are very close from a taxonomic point of view when phylogenetic tree was constructed on the basis of endoglucanase gene sequences of different species of Genus *Bacillus* (Fig. 3). Attempt to detect *B. subtilis* directly from soil samples collected from agriculture field and fish culture ponds were successful, out of 24 soil samples collected from fish pond and agricultural field ten samples were positive for amplification. Cloning and sequencing confirmed the amplicon to be endoglucanase gene of *B. subtilis*. However, after enrichment of negative soil samples on TSB 20 % increased positivity rate was obtained by PCR, demonstrating that the initial concentration of *B. subtilis* was at a proportion below the detection limit. The primers not only differentiated *B. subtilis* from other species but also differentiated at subspecies level as expected product size could not be predicted from *B. subtilis* subsp. *spizizenii* by primer blast (NCBI).

In this report, a PCR method has been established for identification of *B. subtilis.* Endo-β-1,4-glucanase gene has been chosen to design primers that could be useful for identification and direct detection of *B. subtilis* from environmental samples. Detection of *B. subtilis* has been shown to be specific, although the primers showed specificity for *G. stearothermophilus* and *P. campinasensis* in primer blast, and predicted amplicon to be 1,311 bp. However, *B. subtilis* could be differentiated from *G. stearothermophilus* and *P. campinasensis* by sequencing of the pcr product. 16S rRNA gene sequence analysis is the most commonly used method for identifying bacteria or for constructing bacterial phylogenetic relationships (Woese 1987; Vandamme et al. 1996; Joung and Cote 2002); however, its usefulness is limited because of the high percentage of sequence similarity between closely related species (Ash et al. 1991; Marti’nez-Murcia et al. 1992; Christensen et al. 1998). The use of protein-encoding genes as phylogenetic markers is now a common approach (Yamamoto and Harayama 1998; Ko et al. 2004; Chelo et al. 2007). Detailed investigations have demonstrated that sequences from protein-encoding genes can accurately predict genome relatedness and may replace DNA–DNA hybridization for species identification and delineation in the future (Stackebrandt et al. 2002; Zeigler 2003). Wang et al. (2007) clearly showed that in the *B. subtilis* group, within which species differentiation is very difficult, core genes such as gyrB allow differentiation on genetic basis. Compared to other genera, *Bacillus* species are having conserved 16s rRNA sequences and are difficult to identify at species level using this marker (Stackebrandt and Goebel 1994).

## Conclusion

In the present study, the demonstrated specificity of the oligonucleotides used as PCR primers and results of experiments with soil samples provide the basis to develop a diagnostic assay for identification and detection *B. subtilis* form environment samples. As the primers used in this study have been found to be specific to endoglucanase gene of *G. stearothermophilus* and *P. campinasensis,* we suggest to use these primers as supplementary PCR assay to 16s rRNA sequencing for identification of *B. subtilis.*
